# Mitochondrial reactive oxygen species enhance AMP-activated protein kinase activation in the endothelium of patients with coronary artery disease and diabetes

**DOI:** 10.1042/CS20120239

**Published:** 2012-11-27

**Authors:** Ruth M. Mackenzie, Ian P. Salt, William H. Miller, Angela Logan, Hagar A. Ibrahim, Andrea Degasperi, Jane A. Dymott, Carlene A. Hamilton, Michael P. Murphy, Christian Delles, Anna F. Dominiczak

**Affiliations:** *Institute of Cardiovascular and Medical Sciences, College of Medical, Veterinary and Life Sciences, University of Glasgow, Glasgow, U.K.; †MRC Mitochondrial Biology Unit, Cambridge, U.K.; ‡School of Computing Science, University of Glasgow, Glasgow, U.K.

**Keywords:** AMP-activated protein kinase (AMPK), coronary artery disease (CAD), diabetes, endothelium, mitochondrion, oxidative stress, AICAR, 5-amino-4-imidazolecarboxamide riboside, AMPK, AMP-activated protein kinase, BMI, body mass index, CABG, coronary artery bypass graft, CAD, coronary artery disease, CaMKK, Ca^2+^/calmodulin-dependent kinase kinase, CVD, cardiovascular disease, 2DG, 2-deoxy-D-glucose, DTPP, decyl triphenylphosphonium bromide, eNOS, endothelial nitric oxide synthase, *GAPDH*, encoding glyceraldehyde-3-phosphate dehydrogenase, HbA_1c_, glycated haemoglobin, HDL, high-density lipoprotein, HSVEC, human saphenous vein endothelial cell, HUVEC, human umbilical vein endothelial cell, LDL, low-density lipoprotein, ROS, reactive oxygen species, mtROS, mitochondrial ROS, *PRKAA1*, encoding the AMPK-α1 catalytic subunit, SOD, superoxide dismutase, T2D, Type 2 diabetes, vWF, von Willebrand factor

## Abstract

The aim of the present study was to determine whether the endothelial dysfunction associated with CAD (coronary artery disease) and T2D (Type 2 diabetes mellitus) is concomitant with elevated mtROS (mitochondrial reactive oxygen species) production in the endothelium and establish if this, in turn, regulates the activity of endothelial AMPK (AMP-activated protein kinase). We investigated endothelial function, mtROS production and AMPK activation in saphenous veins from patients with advanced CAD. Endothelium-dependent vasodilation was impaired in patients with CAD and T2D relative to those with CAD alone. Levels of mitochondrial H_2_O_2_ and activity of AMPK were significantly elevated in primary HSVECs (human saphenous vein endothelial cells) from patients with CAD and T2D compared with those from patients with CAD alone. Incubation with the mitochondria-targeted antioxidant, MitoQ_10_ significantly reduced AMPK activity in HSVECs from patients with CAD and T2D but not in cells from patients with CAD alone. Elevated mtROS production in the endothelium of patients with CAD and T2D increases AMPK activation, supporting a role for the kinase in defence against oxidative stress. Further investigation is required to determine whether pharmacological activators of AMPK will prove beneficial in the attenuation of endothelial dysfunction in patients with CAD and T2D.

## INTRODUCTION

The prevalence of CAD (coronary artery disease) in patients with diabetes is notably higher than in the general population. Up to a third of patients requiring coronary intervention have diabetes, and outcome is poorer in these patients than in patients without diabetes [1,2]. With the worldwide incidence and prevalence of diabetes increasing, more individuals will be affected by CAD and related complications and further pressure on health systems is expected [3]. The relationship between diabetes and vascular disease is complex and multifactorial [1]. Increased vascular oxidative stress has been proposed as a mechanism responsible for impaired endothelial function in patients with diabetes [4], but it is unknown whether these findings persist in the light of the more aggressive primary and secondary prevention strategies to which these vulnerable patients are subject [3].

Mitochondria are a major source of ROS (reactive oxygen species) production in the vasculature and contribute to oxidative stress and endothelial dysfunction in a range of cardiovascular pathologies, including CAD and T2D (Type 2 diabetes mellitus) [5,6]. However, mtROS (mitochondrial ROS) are also of importance in cellular signalling and, at low O_2_ concentrations, mitochondria of HUVECs (human umbilical vein endothelial cells) have been shown to generate ROS for activation of enzymes such as AMPK (AMP-activated protein kinase) [7].

Human AMPK is a heterotrimeric serine/threonine kinase, consisting of a catalytic α-subunit and regulatory β- and γ-subunits, each of which has two or more isoforms that are encoded by distinct genes and differentially expressed in various tissues [8]. Involved in the regulation of cellular and whole body energy status [9], activation of AMPK requires phosphorylation at Thr^172^ by an AMPK kinase. Two AMPK kinases have been isolated to date, LKB1 [10] and CaMKK (Ca^2+^/calmodulin-dependent kinase kinase) [11]. It has been proposed that LKB1 activity is constitutive, such that stimuli which increase the AMP/ATP ratio, including hypoxia, hypoglycaemia and ischaemia, inhibit dephosphorylation at Thr^172^, permitting phosphorylation and activation of AMPK by LKB1 [10]. In contrast, activation of AMPK by Ca^2+^ is AMP-independent and mediated by CaMKK [11,12]. More recently, AMPK activation via a ROS-mediated mechanism has been described [7,13–18].

Proposed as a candidate target for therapeutic intervention in endothelial dysfunction, AMPK stimulates eNOS (endothelial nitric oxide synthase), leading to NO production in cultured endothelial cells [19]. Furthermore, stimulation of endothelial AMPK is reported to attenuate pro-inflammatory signalling [20,21] and there is accumulating evidence to suggest a role for the kinase in defence against oxidative stress in the endothelium [8,22,23].

The present study was designed to test the hypothesis that the endothelial dysfunction associated with CAD and T2D occurs in parallel with increased mtROS production in the endothelium and that this, in turn, regulates endothelial AMPK activity.

## MATERIALS AND METHODS

Detailed methods can be found in Supplementary Materials and methods section at http://www.clinsci.org/cs/124/cs1240403add.htm.

### Subjects

We recruited 79 volunteers with severe CAD from cardiothoracic pre-operative clinics. A blood sample was taken after 3 h of fasting on the day of admission for CABG (coronary artery bypass graft) surgery. Twenty three volunteers had a history of T2D. T2D was defined as having fasting venous blood glucose ≥6.1 mmol/l or ≥10 mmol/l 2 h post-oral glucose load (75 g). We also recruited 19 control volunteers free of evidence of CAD who were undergoing surgery for the removal of varicose veins. For these volunteers, a blood sample was taken following 3 h of fasting 1–2 weeks after surgery. The study complies with the Declaration of Helsinki and was approved by the local ethics committee. All participants gave written informed consent.

### Preparation of vascular tissue

Surplus portions of freshly harvested saphenous veins from volunteers undergoing CABG surgery or elective varicose vein removal were stored in sterile saline solution. Maximum storage time was 2 h. Only non-varicosed portions of veins from control patients, as identified by the surgical team, were utilized. Samples were taken to the laboratory and cleaned of excess connective tissue. Endothelial cells were isolated from sections of samples on the day of surgery and the remainder of samples stored at 4°C in a Krebs Hepes buffer (118 mmol/l NaCl, 10 mmol/l Hepes/NaOH, pH 7.4, 25 mmol/l NaHCO_3_, 4.7 mmol/l KCl, 1.2 mmol/l MgSO_4_, 1.2 mmol/l KH_2_PO_4_, 11 mmol/l glucose, 10 μmol/l indomethacin and 50 μmol/l EDTA) for study of endothelial function the following day.

### Assessment of endothelial function

Rings (3 mm) of saphenous vein were studied in organ bath chambers as previously described [24]. Vessels were constricted with phenylephrine (3 μmol/l) and relaxation in response to the calcium ionophore A23187 (0.01–10 μmol/l) was studied. Maximum relaxation was calculated and is expressed as a percentage of constriction in response to phenylephrine.

### Cell culture

HSVECs (human saphenous vein endothelial cells) were isolated on the day of surgery by standard collagenase digestion based on a modified version of the protocol described by Jaffe et al. [25]. Cells were cultured in complete Large Vessel Endothelial Cell Basal Medium (TCS Cellworks), supplemented with 20% (v/v) FBS (fetal bovine serum), 100 units/ml penicillin, 100 μg/ml streptomycin and 2 mmol/l L-glutamine. Cells were used at passage 3 and all experimental procedures were carried out when cells were about 80% confluent.

For immunofluorescent staining of vWF (von Willebrand factor), passage 3 HSVECs were harvested and plated onto sterile coverslips before being fixed in 4% (w/v) PFA (paraformaldehyde). Cells were then incubated with mouse anti-vWF primary antibody [1:50 dilution in 20% (v/v) goat serum/PBS] followed by goat-anti-(mouse IgG)–FITC-conjugated secondary antibody (1:200 dlution in 20% (v/v) goat serum/PBS; Dako). Coverslips were subsequently mounted in Vectashield (Vector Laboratories), containing propidium iodide for nucleic counter-staining, and cells visualized under a fluorescence microscope (Olympus BX40).

### Assessment of mitochondrial H_2_O_2_ production

Measurement of HSVEC mitochondrial H_2_O_2_ production was carried out using the mitochondria-targeted H_2_O_2_ MS probe, MitoB, as described previously for cell culture experiments [26]. Where required, HSVECs were incubated at 37°C in medium supplemented with 1 μmol/l MitoQ_10_ for 1 h prior to washing in PBS and incubation in medium supplemented with 5 μmol/l MitoB for 6 h.

### mRNA expression

Total RNA was extracted from HSVECs using the RNeasy® Mini Kit (QIAGEN) and quantified (NanoDrop ND-100 Spectrophotometer). cDNA was synthesized from 1 μg of DNase-treated (TURBO DNA-free™; Ambion) total RNA (TaqMan® Reverse Transcription Reagents; Applied Biosystems). Relative quantification of *PRKAA1* (encoding the AMPK-α1 catalytic subunit) expression, relative to *GAPDH* (encoding glyceraldehyde-3-phosphate dehydrogenase), was calculated using the comparative (ΔΔ*C*_t_) method [27].

### AMPK activity assay

HSVECs were serum-starved overnight before being incubated for 1 h at 37°C in Krebs Ringer Hepes buffer (119 mmol/l NaCl, 20 mmol/l Hepes, pH 7.4, 5 mmol/l NaHCO_3_, 4.7 mmol/l KCl, 1.3 mmol/l CaCl_2_, 1.2 mmol/l MgSO_4_, 1 mmol/l KH_2_PO_4_, 0.1 mmol/l L-arginine, 5 mmol/l glucose) in the presence of 1 μmol/l MitoQ_10_, 1 μmol/l DTPP (decyl triphenylphosphonium bromide) and 2 mmol/l AICAR (5-amino-4-imidazolecarboxamide riboside) where required. Cell lysates were prepared and AMPK immunoprecipitated and assayed using the SAMS peptide as previously described [28].

### Statistical analyses

For clinical data and measurements in whole vessels, continuous data are given as means±S.D. or medians (interquartile range) unless otherwise indicated. Values stated are means±S.E.M. for cellular data. For comparisons of a continuous variable between two experimental groups, paired and unpaired Student's *t* test and Mann–Whitney *U* tests were applied as appropriate. For comparisons of a continuous variable in datasets with more than two groups, ANOVA was applied, followed by the Tukey's post-hoc test for all possible pairwise comparisons. Categorical data were analysed by Fisher's exact test. A *P* value of less than 0.05 (two-tailed) was considered significant.

## RESULTS

### Characteristics of study participants

Demographic and clinical characteristics of patients and control subjects are given in Table 1. As expected, patients with CAD were older and more likely to be on cardiovascular medication than control subjects. Total cholesterol and LDL (low-density lipoprotein)-cholesterol levels were lower in patients with CAD compared with control subjects, consistent with lipid-lowering therapy in the patient group. HDL (high-density lipoprotein)-cholesterol levels were significantly greater in control subjects compared with patients. Patients with T2D had a greater BMI (body mass index) and a greater percentage of HbA_1c_ (glycated haemoglobin), but no other significant differences to patients without diabetes were observed. A total of ten out of the 23 patients with T2D (43%) were treated with metformin.

**Table 1:** Characteristics of the study cohort Continuous data are given as means±S.D., irrespective of distribution or skewness. *P* values, however, derive from a Student's *t* test or Mann–Whitney *U* test as appropriate. Comparison between categorical data was performed using Fisher's exact test. SBP, systolic blood pressure; DBP, diastolic blood pressure; TAG, triacylglycerol; CRP, C-reactive protein; HbA_1c_, glycosylated haemoglobin; ACEI, angiotensin-converting enzyme inhibitor; ARB, angiotensin II type I receptor blocker. *With T2D compared with Without T2D; †CAD compared with controls.

	CAD patients				
Parameter	With T2D (*n*=23)	Without T2D (*n*=56)	*P* value*	Controls (*n*=19)	*P* value†
Age (years)	65±11	65±9	0.961	44±19	<0.001
Sex (male/female) (*n*)	20/3	45/11	0.747	8/11	0.001
BMI (kg/m^2^)	31.7±5.2	28.6±4.7	0.026	26.5±1.9	0.004
SBP (mmHg)	138±22	138±26	0.897	132±23	0.471
DBP (mmHg)	73±13	80±11	0.065	84±12	0.162
Total cholesterol (mmol/l)	3.95±1.10	4.11±0.99	0.542	4.96±0.99	0.029
LDL-cholesterol (mmol/l)	1.80±0.83	2.00±0.78	0.314	2.69±1.11	0.024
HDL-cholesterol (mmol/l)	1.11±0.22	1.17±0.27	0.366	1.72±0.30	<0.001
TAG (mmol/l)	2.54±1.97	2.04±0.97	0.262	1.17±0.47	0.052
CRP (mg/l)	2.5±2.9	5.0±10.5	0.280	3.5±4.2	0.843
HbA_1c_ (%)	7.2±1.3	5.6±0.4	<0.001	5.4±0.2	0.111
Active smoking (yes/no) (*n*)	2/22	2/54	0.579	2/17	0.324
ACEI/ARB (yes/no) (*n*)	18/5	32/24	0.122	1/18	<0.001
Statin (yes/no) (*n*)	21/2	53/3	0.625	2/17	<0.001
Metformin (yes/no) (*n*)	10/13	0/56	<0.001	0/19	<0.001

### Endothelial function

**Figure 1:**
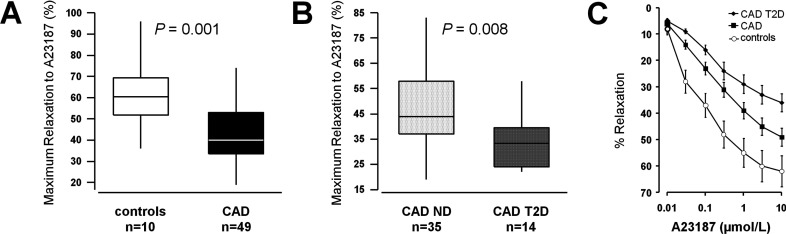
Vasorelaxation in human saphenous veins Maximum relaxation of saphenous vein in response to the endothelium-dependent vasodilator calcium ionophore A23187 (**A**, **B**). (**A**) Comparisons between patients with CAD (*n*=49) and control subjects (*n*=10). (**B**) Comparisons between patients with CAD and T2D (*n*=14) and patients with CAD alone (CAD ND, *n*=35). (**C**) Dose–response curves to A23187 for the three groups (means±S.E.M.).

Endothelium-dependent relaxation was impaired in vessels of patients with CAD compared with those obtained from control subjects (maximum relaxation in response to A23187, 43±16 compared with 62±16%; *P*=0.001; Figures 1A and 1C). Patients with CAD and T2D had significantly reduced endothelium-dependent relaxation compared with those with CAD alone (maximum relaxation in response to A23187, 34±11 compared with 47±16%; *P*=0.008; Figures 1B and 1C). See Supplementary Figure S1 (at http://www.clinsci.org/cs/124/cs1240403add.htm) for data in saphenous veins concerning the vasorelaxation in response to carbachol and Supplementary Figures S1 and S2 (at http://www.clinsci.org/cs/124/cs1240403add.htm) for endothelium-dependence of the relaxation in response to carbachol and A23187.

### Mitochondrial H_2_O_2_ production in primary HSVECs

**Figure 2:**
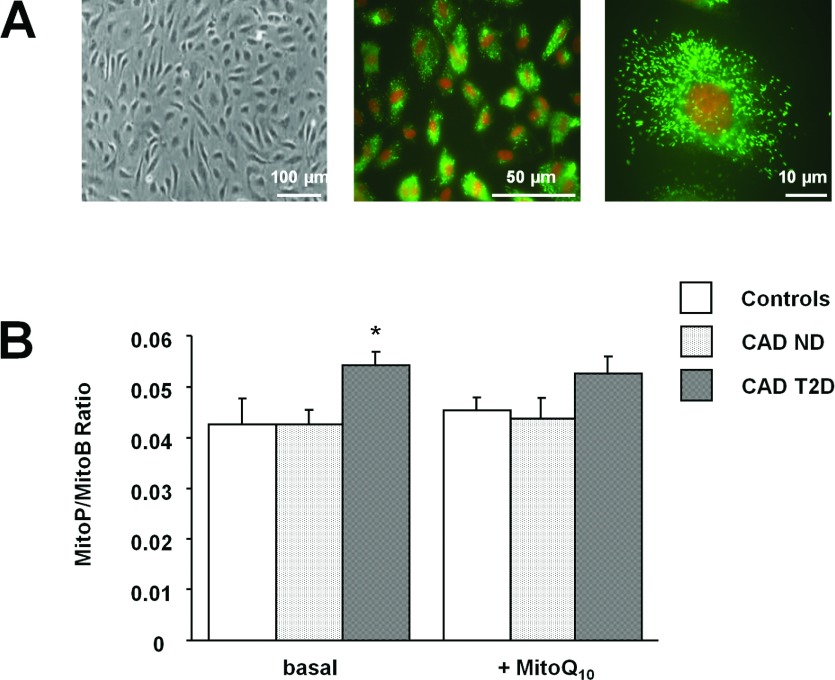
Mitochondrial H_2_O_2_ production in HSVECs (**A**) Representative photomicrographs showing HSVECs in primary culture. Endothelial cells were successfully isolated, as determined by their characteristic cobblestone morphology (left-hand panel) and positive staining (green) for vWF (middle and right-hand panels). (**B**) HSVEC mitochondrial H_2_O_2_ production was determined using the mitochondria-targeted MS probe, MitoB, in both the presence and absence of the mitochondria-targeted antioxidant, MitoQ_10_. *P<0.05 compared with CAD ND. CAD ND, CAD patients without T2D (*n*=4); CAD T2D, CAD patients with T2D (*n*=4); controls, control subjects (*n*=3).

It has been demonstrated that mtROS contribute to the oxidative stress and endothelial dysfunction characteristic of CVD (cardiovascular disease) [5,6]. To establish whether increased levels of mitochondrially produced H_2_O_2_ were concomitant with the endothelial dysfunction observed in vessels from patients with CAD, we investigated mitochondrial H_2_O_2_ production in primary HSVECs (Figure 2A) isolated from these patients and control subjects.

Significantly greater mitochondrial H_2_O_2_ production was noted in HSVECs from CAD patients with T2D relative to those from patients with CAD alone (MitoP/MitoB ratio, 0.054±0.003 compared with 0.042±0.003; *P*=0.02; Figure 2B). This finding was independent of mitochondria number (Supplementary Figure S3 at http://www.clinsci.org/cs/124/cs1240403add.htm). The mitochondrial-targeted antioxidant MitoQ_10_ had no significant effect on basal mitochondrial H_2_O_2_ production in HSVECs from patients with CAD alone (MitoP/MitoB ratio, 0.042±0.003 compared with 0.044±0.004; *P*=0.61; Figure 2B), patients with CAD and T2D (MitoP/MitoB ratio, 0.054±0.003 compared with 0.052±0.004; *P*=0.35; Figure 2B) and control subjects (MitoP/MitoB ratio, 0.043±0.006 compared with 0.045±0.003, *P*=0.58; Figure 2B).

### AMPK activation in primary HSVECs

AMPK has recently been implicated in attenuation of endothelial oxidative stress [8,22,23]. In addition, the kinase has been shown to be activated in response to a variety of ROS, including H_2_O_2_ [13,18,29].

**Figure 3:**
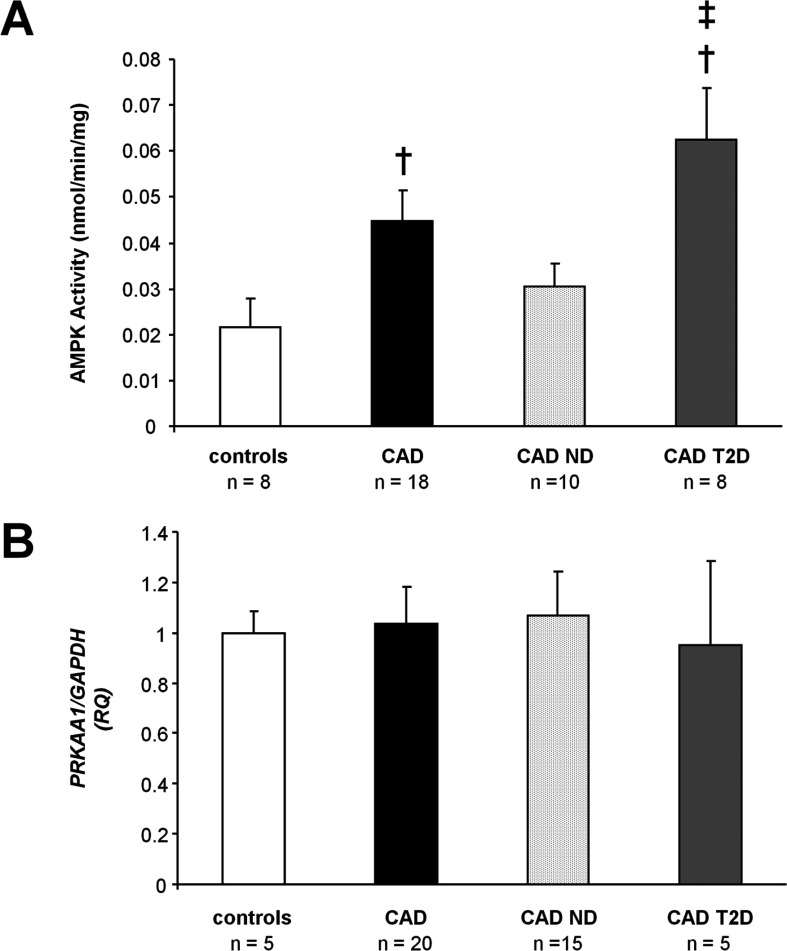
AMPK activation in HSVECs (**A**) Total AMPK activity in immunoprecipitates from HSVEC lysates cultured at 21% O_2_. †*P*<0.01 compared with control subjects, ‡*P*<0.01 compared with CAD ND. (**B**) HSVEC *PRKAA1* mRNA expression relative to *GAPDH*. RQ, relative quantification; CAD ND, CAD patients without T2D; CAD T2D, CAD patients with T2D.

As shown in Figure 3(A), activity of AMPK was increased in cells from CAD patients as compared with those from control subjects (0.045±0.007 compared with 0.022±0.006 nmol·min^−1^·mg^−1^; *P*=0.05). When compared with control subjects, a greater increase in AMPK activity was observed in HSVECs from patients with CAD and T2D (0.062±0.011 compared with 0.022±0.006 nmol·min^−1^·mg^−1^; *P*=0.01; Figure 3A) than in cells from patients with CAD alone (0.031±0.005 compared with 0.022±0.006 nmol·min^−1^·mg^−1^; *P*=0.26; Figure 3A). AMPK activity was significantly higher in HSVECs from patients with CAD and T2D than in those from patients with CAD alone (0.062±0.011 compared with 0.031±0.005; *P*=0.01; Figure 3A).

No change in the mRNA expression of *PRKAA1*, encoding the AMPK-α1 catalytic subunit, was observed between patient groups (Figure 3B), consistent with modulation of AMPK activity via post-translational modification. In addition, there was no significant difference in protein expression of AMPK-α1 (Supplementary Figure S4 at http://www.clinsci.org/cs/124/cs1240403add.htm) or the upstream AMPK kinase LKB1 (Supplementary Figure S5 at http://www.clinsci.org/cs/124/cs1240403add.htm), as assessed by immunoblotting.

### AMPK substrate phosphorylation in primary HSVECs

**Figure 4:**
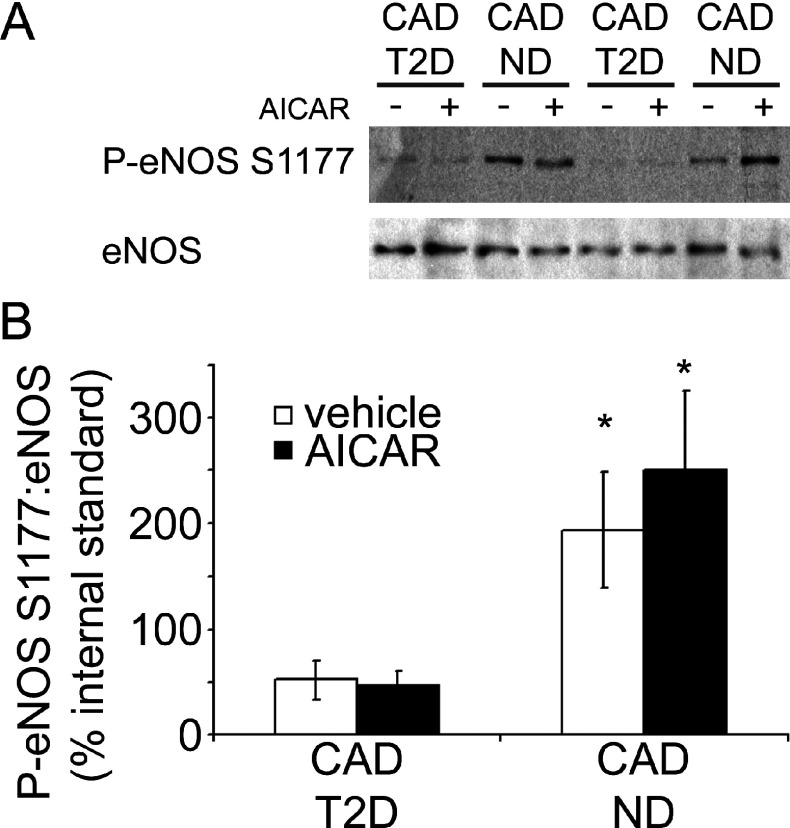
Comparison of eNOS Ser^1177^ phosphorylation in HSVECs HSVECs were isolated from CAD patients with (CAD T2D, *n*=5) and without (CAD ND, *n*=5) T2D and eNOS Ser^1177^ phosphorylation in the presence or absence of AICAR assessed by Western blotting of cell lysates. (**A**) Representative immunoblots from two CAD patients with and two CAD patients without T2D. (**B**) Densitometric analysis of the P-eNOS (phospho-eNOS Ser^1177^)/eNOS ratio relative to an internal standard lysate.*P<0.05 compared with CAD T2D (vehicle).

Given the increased AMPK activity observed in HSVECs from patients with CAD and the poorer endothelial function of these subjects, phosphorylation of eNOS, an AMPK substrate, was investigated in cells from these CAD patients by means of immunoblotting (Figure 4A). Densitometric analysis revealed significantly lower basal eNOS phosphorylation in HSVECs from CAD patients with T2D (Figure 4B), despite the increased basal AMPK activity in these cells (Figure 3A). Incubation of HSVECs with AICAR, an artificial activator of AMPK [30], appeared to result in increased eNOS phosphorylation in cells from patients with CAD alone, although results were not significant (Figure 4B). However, AICAR treatment failed to stimulate eNOS phosphorylation in HSVECs from those CAD patients with T2D (Figure 4B).

### Mitochondrial ROS-mediated AMPK activation in primary HSVECs

Vascular endothelial cells have been reported to be highly glycolytic [7]. In support of this, HSVEC ATP synthesis was largely the result of glycolysis, as assessed by the relative effects of 2DG (2-deoxy-D-glucose) and rotenone (Supplementary Figure S6 at http://www.clinsci.org/cs/124/cs1240403add.htm).

**Figure 5:**
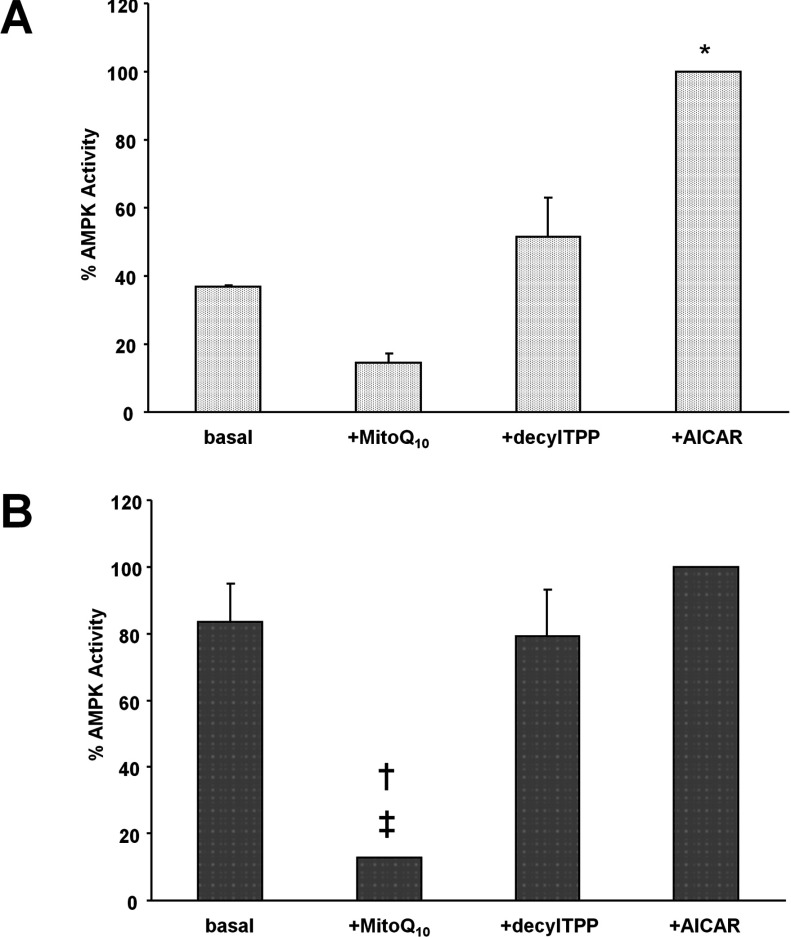
Effect of mitochondrial-ROS on AMPK activation in HSVECs (**A**) Cells from CAD patients without T2D (CAD ND, *n*=5) and (**B**) with T2D (CAD T2D, *n*=5) were incubated in the presence (+) of MitoQ_10_, DTPP and AICAR. Total AMPK was immunoprecipitated from lysates which were then assayed for AMPK activity.*P=0.05 compared with basal value for CAD ND; †*P*<0.05 compared with basal value for CAD T2D; ‡*P*<0.01 compared with +DTPP value for CAD T2D.

An alternative, potentially significant role for endothelial mitochondria is the generation of ROS for signalling purposes [7]. The contribution of mtROS to AMPK activation was investigated by treating HSVECs from CAD patients with MitoQ_10_ and subsequently assaying AMPK activity. To control for non-specific effects of MitoQ_10_, cells were treated with the non-active control compound, DTPP [31]. Exposing HSVECs to MitoQ_10_ resulted in a reduction in AMPK activity (Figure 5). The MitoQ_10_-mediated decrease in AMPK activity was greater in HSVECs from CAD patients with T2D (0.056±0.004 compared with 0.009±0.01 nmol·min^−1^·mg^−1^; *P*=0.02; Figure 5B) than in those from patients with CAD alone (0.035±0.005 compared with 0.014±0.0002 nmol·min^−1^·mg^−1^; *P*=0.06; Figure 5A).

Treating HSVECs from CAD patients with AICAR resulted in a significant increase in kinase activity in cells from patients with CAD alone (0.035±0.005 compared with 0.095±0.01 nmol·min^−1^·mg^−1^; *P*=0.05; Figure 5A).

## DISCUSSION

Increased vascular oxidative stress has been proposed as one potential mechanism underlying endothelial dysfunction in patients with CAD and T2D [4]. The present study involved investigation of endothelial function and molecular determinants of oxidative stress in human saphenous veins. Previous studies have demonstrated human arterial and venous levels of oxidative stress are closely related [32,33], such that results should not be affected by the choice of vessel. Herein we report impaired endothelium-dependent relaxation in vessels from patients with advanced CAD. Poorer endothelial function was observed in those CAD patients with the additional cardiovascular risk factor, T2D. To investigate the molecular basis for the impaired endothelium-dependent relaxation in vessels from patients with CAD and T2D, endothelial cells were isolated from vascular tissue. Endothelial dysfunction was observed to be maintained in culture with significantly lower levels of basal eNOS Ser^1177^ phosphorylation in cells from CAD patients with T2D as compared with those with CAD alone. As isolated cells were maintained in culture for several weeks prior to investigation, it seemed likely that the effects of pharmacological treatments (including metformin, a known activator of AMPK [8]) would be lost, allowing more accurate insight into molecular determinants of impaired endothelial function and potentially causative oxidative stress.

The mitochondrial electron transport chain has been identified as a major source of ROS in the vasculature, contributing to the oxidative stress and endothelial dysfunction characteristic of CAD and T2D [5,6]. As such, following isolation and characterization of HSVECs, we investigated cellular mitochondrial H_2_O_2_ production and observed an increase in cells from patients with CAD and T2D which was not due to an increase in mitochondria number. Known to induce endothelial dysfunction [34], increased levels of mitochondrially produced H_2_O_2_ may therefore have a causal role in the significantly impaired vasorelaxation of CAD patients with T2D.

Vascular endothelial cells are recognized as being highly glycolytic and we have confirmed this to be the case for HSVECs. A potential reason for the favouring of glycolysis by endothelial cells has been proposed by Quintero and co-workers [7], whereby mitochondria are not preferentially used bioenergetically in these cells, allowing them to function primarily in the generation of ROS for signalling purposes, resulting in activation of enzymes, including AMPK.

Traditionally associated with maintenance of cellular energy homoeostasis, it is well documented that AMPK is activated in response to the increased AMP/ATP ratio characteristic of hypoxic stress. With regard to endothelial AMPK specifically, phosphorylation of the kinase has been observed at low O_2_ concentrations in HUVECs but is undetectable at 21% O_2_ or ‘normoxia’ [7]. However, it has been suggested that kinase activation at low O_2_ concentrations may occur via a mechanism that is independent of altered nucleotide levels but is mtROS-mediated [7,13]. Interestingly, we were able to assay AMPK activity in HSVECs isolated from patients with CAD and cultured under normoxic conditions, indicating CVD phenotype could be linked to enzyme activation. Indeed, at 21% O_2,_ HSVEC AMPK activity was significantly greater in cells from patients with CAD relative to control subjects. On stratifying CAD patients according to the presence of T2D, we found AMPK activity to be significantly increased in the endothelium of patients with CAD and T2D as compared with that of patients with CAD alone, despite no change in AMPKα1 expression or difference in levels of the upstream AMPK kinase LKB1. Incubating HSVECs with AICAR, an artificial, ROS-independent activator of AMPK, known to stimulate the kinase in endothelial cells [19], resulted in a significant increase in AMPK activity in cells from patients with CAD alone but not in cells from those patients with CAD and T2D whose basal AMPK activity approached maximal levels.

Given the elevated mitochondrial H_2_O_2_ production in HSVECs from patients with CAD and T2D, in addition to the glycolytic nature of the cells, it seemed likely that enhanced endothelial AMPK activation was occurring in an mtROS-mediated manner in these patients. In order to test this hypothesis, we treated HSVECs isolated from CAD patients with the mitochondria-targeted antioxidant, MitoQ_10_, which has been shown to prevent oxidative damage in endothelial cells *in vitro* [35]. Our findings demonstrated a significant decrease in AMPK activation on treatment with MitoQ_10_ in cells from those patients with T2D. The non-antioxidant control for MitoQ_10_, DTPP, had no effect on AMPK activity in a parallel experiment, indicating results can be attributed to the antioxidant action of MitoQ_10_ specifically_._ The same effect was not seen in cells from CAD patients without T2D.

Taken together, our findings indicate a novel, mtROS-mediated activation of AMPK in the endothelium of patients with CAD and T2D. In terms of mtROS likely to be involved in activation of the kinase, in concordance with results presented here, a role for H_2_O_2_ has been reported [13,18,29]. However, MitoQ_10_ does not act by directly lowering H_2_O_2_ production [36], confirmed via investigation of HSVEC mitochondrial H_2_O_2_ levels in the presence and absence of the antioxidant. Therefore the signal emanating from mitochondria and activating AMPK in the endothelium of patients with CAD and T2D is unlikely to be H_2_O_2_ itself, but rather a downstream radical with which MitoQ_10_ reacts. Such radicals include lipid peroxidation products, generated on oxidation of mitochondrial lipids by H_2_O_2_.

Recent studies suggest AMPK activation improves endothelial function by counteracting oxidative stress in the endothelium. Indeed, the kinase suppresses NADPH oxidase and ROS production in endothelial cells [23] and stimulates NO production by eNOS, inducing endothelium-dependent vasodilation [37]. In addition, AMPK activation attenuates pro-inflammatory signalling and monocyte adhesion to the endothelium [20]. Furthermore, metformin, known to exert a portion of its effect through AMPK, has been reported to decrease intracellular production of mtROS in aortic endothelial cells [38], while activation of AMPK has been observed to reduce hyperglycaemia-induced mtROS production by induction of the endogenous mitochondrial antioxidant, SOD2 (superoxide dismutase 2) in HUVECs [39]. Similarly, Colombo and Moncada [22] have demonstrated that endothelial AMPKα1 is responsible for the expression of a number of genes involved in antioxidant defence, including *SOD2*.

Our observations that eNOS Ser^1177^ phosphorylation is significantly reduced in cells from CAD patients with T2D implies that elevated AMPK activity alone against a background of T2D is not sufficient to increase eNOS phosphorylation at this residue. It could perhaps be the case that a phosphatase is activated in these patients or that this additional CVD risk factor results in eNOS being regulated in an alternative manner, rendering it much more difficult to phosphorylate. Interestingly, AMPK has recently been shown to phosphorylate eNOS at the additional residue Ser^633^ and ablation of AMPKα2 was observed to be sufficient to inhibit atorvasatin-stimulated eNOS phosphorylation at both this residue and Ser^1177^, despite AMPKα1 being the principle isoform in terms of total cellular activity [40]. Consequently, it is feasible that the increased AMPK activity observed in CAD patients with T2D reflects increased AMPKα1 activity alone and that AMPKα2 activity is required for eNOS Ser^1177^ phosphorylation. In accordance with our findings, Wang et al. [41] have reported that HUVECs subject to low glucose concentrations demonstrate reduced NO bioavailability associated with increased mtROS production and AMPK activation, yet this fails to stimulate eNOS phosphorylation.

**Figure 6:**
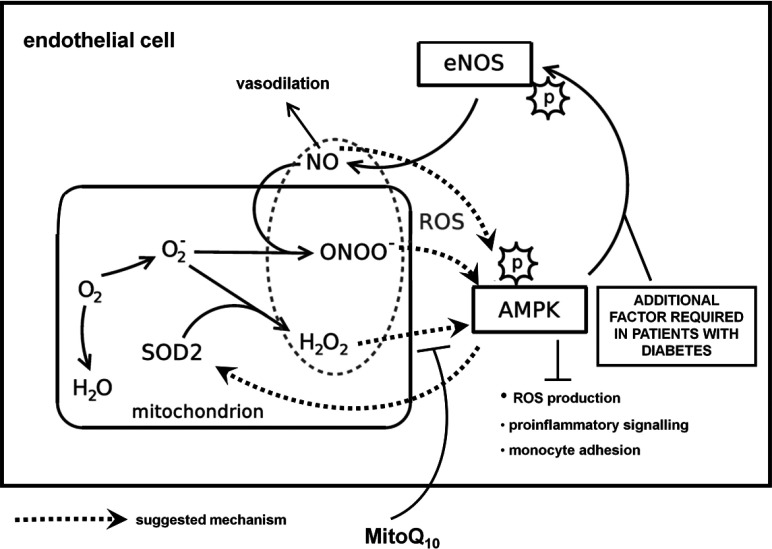
Proposed mechanism of mtROS-mediated AMPK activation in endothelial cells The results suggest a potentially mtROS-mediated increase in AMPK activity in patients with CAD and T2D. The mtROS in question are likely to be downstream derivates of H_2_O_2_, such as lipid peroxidation products. AMPK may therefore be part of a feedback or adaptive mechanism with a role in defence against oxidative stress in the endothelium, attenuating pro-inflammatory signalling and regulating expression of antioxidant genes, including *SOD2*. However, increased kinase activity does not appear to be sufficient to stimulate activation of eNOS, in patients with T2D.

In the endothelium of patients with CAD and T2D, AMPK may be part of a feedback or adaptive mechanism, wherein elevated mtROS production results in activation of AMPK which, in turn, stimulates protective responses which may contribute towards, but are not solely responsible for, increasing NO bioavailability and attenuating endothelial dysfunction. Although not investigated here, the mtROS-activated AMPK may also induce SOD2 activity, thus counteracting mitochondrial oxidative stress and generating H_2_O_2_ for further kinase activation and perpetuation of the cycle (Figure 6).

In summary, our results demonstrate elevated mtROS production in the endothelium of patients with CAD and T2D, suggesting mitochondria contribute to the more severe endothelial dysfunction observed in these patients. In addition, we have shown a novel, mtROS-mediated mechanism for AMPK activation in the endothelium of patients with CAD and T2D. Although this novel AMPK activation supports a role for the kinase in counteraction of oxidative stress, we demonstrate that increased AMPK activity does not simply translate to increased eNOS phosphorylation in these subjects and is therefore not sufficient to attenuate the more severe endothelial dysfunction characteristic of diabetic patients.

## CLINICAL PERSPECTIVES

• Increasing evidence exists to suggest that elevated mtROS production may contribute to poorer endothelial function in patients with CAD and T2D compared with patients without T2D.
• In the present study, we have demonstrated significantly increased levels of mtROS in primary endothelial cells from patients with CAD and T2D and a concomitant increase in endothelial AMPK activity. This enhanced kinase activity is, however, insufficient to increase eNOS phosphorylation.
• Further investigation is required to determine whether pharmacological activators of AMPK will prove benefical in attenuating endothelial dysfunction in patients with CAD and T2D.

## Online data

Supplementary data
