# Supplementary material for: Mitochondrial reactive oxygen species enhance AMP-activated protein kinase activation in the endothelium of patients with coronary artery disease and diabetes
Source: Clin Sci (Lond). 2012 Nov 27;124(Pt 6):403–11. doi: 10.1042/CS20120239 (PMC3903000; doi:10.1042/CS20120239)
Supplement: Supplementary data [file cs1240403add.pdf]

Clinical Science (2013) **124**, 403–411 (Printed in Great Britain) doi: 10.1042/CS20120239

Original Paper

www.clinsci.org Clinical Science

SUPPLEMENTARY ONLINE DATA

Mitochondrial reactive oxygen species enhance AMP-activated protein kinase activation in the endothelium of patients with coronary artery disease and diabetes

Ruth M. MACKENZIE*, Ian P. SALT*, William H. MILLER*, Angela LOGAN†, Hagar A. IBRAHIM*, Andrea DEGASPERI‡, Jane A. DYMOTT*, Carlene A. HAMILTON*, Michael P. MURPHY†, Christian DELLES* and Anna F. DOMINICZAK*

*Institute of Cardiovascular and Medical Sciences, College of Medical, Veterinary and Life Sciences, University of Glasgow, Glasgow, U.K.
†MRC Mitochondrial Biology Unit, Cambridge, U.K.
‡School of Computing Science, University of Glasgow, Glasgow, U.K.

MATERIALS AND METHODS

Reagents

Endothelial cell culture medium was obtained from TCS Cellworks (Botolph Claydon) and PromoCell. The mouse anti-vWF antibody was obtained from Dako. Rabbit anti-phospho-AMPK-α1-Thr[172] and anti-LKB1 antibodies were from Cell Signaling Technology, and sheep anti-AMPK-α1 antibody was generously provided by Professor D.G. Hardie (University of Dundee, Dundee, U.K.). 2DG and rotenone were purchased from Sigma. Custom TaqMan® Gene Expression Assays for *PRKAA1* (Hs01562315_m1), *MT-CYB* (mitochondrially encoded cytochrome *b*; Hs02596867_s1) and *HBB* (β-haemoglobin; Hs00758889_s1), and TaqMan® Endogenous Control *GAPDH* (4326317E) were from Applied Biosystems. AICAR was purchased from Toronto Research Chemicals and MitoB, $MitoQ_{10}$ mesylate and DTPP were from Professor Robin Smith (Department of Chemistry, University of Otago, Dunedin, New Zealand). The SAMS peptide (HMRSAMSGLHLVKRR) was synthesized by Pepceuticals.

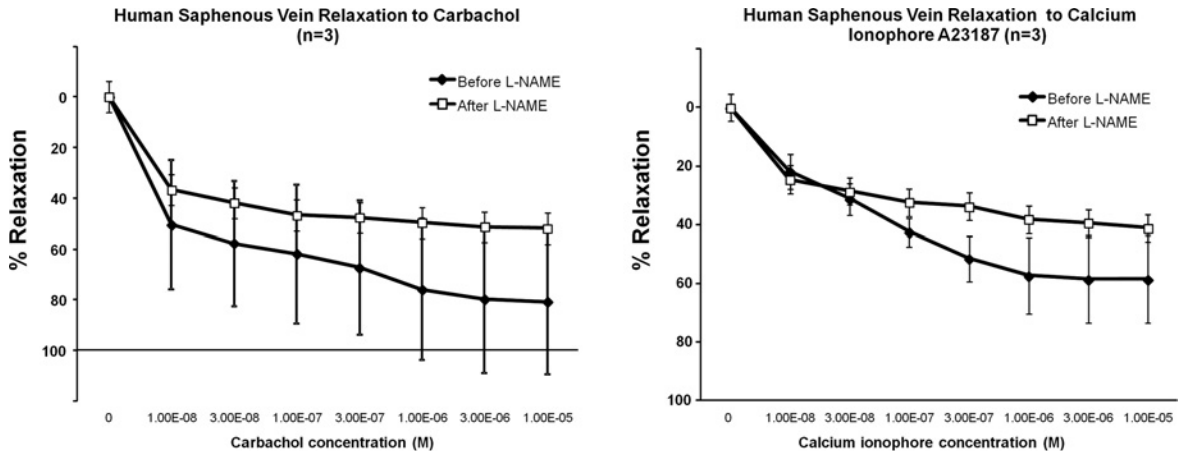

Figure S1 **Vasodilation in response to carbachol and A23187 in saphenous vein samples from patients with CAD but without T2D**
Experiments were performed in saphenous vein samples from three patients with CAD but without T2D. Vasodilation was induced by carbachol (left-hand panel) or calcium ionophore A23187 (right-hand panel). There were no significant differences in the maximum relaxation in response to either compound (filled symbols; 80 % compared with 58 %), but significant blunting of the response after pre-incubation (30 min) with L-NAME (N^G-nitro-L-arginine methyl ester; 0.1 mmol/l; open symbols) in both sets of experiments.

Correspondence: Dr Christian Delles (email Christian.Delles@glasgow.ac.uk).

© 2013 The Author(s)
The author(s) has paid for this article to be freely available under the terms of the Creative Commons Attribution Licence (CC-BY) (http://creativecommons.org/licenses/by/3.0/) which permits unrestricted use, distribution and reproduction in any medium, provided the original work is properly cited.

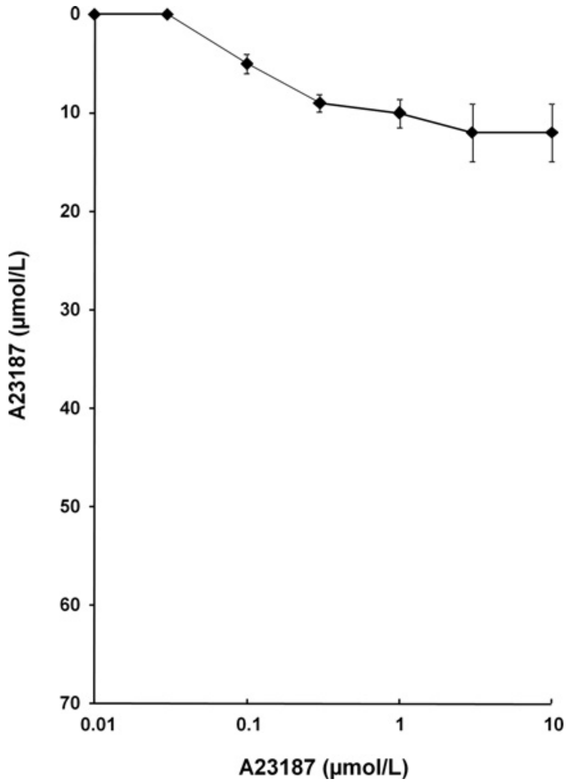

Figure S2 Relaxation in response to the calcium ionophore A23 187 in endothelium-denuded rings of saphenous vein from patients (*n* = 3) with CAD but without T2D

Response to A23187 is significantly blunted compared with experiments in endothelium-intact vessels (see Supplementary Figure S1). Means±S.E.M. are displayed.

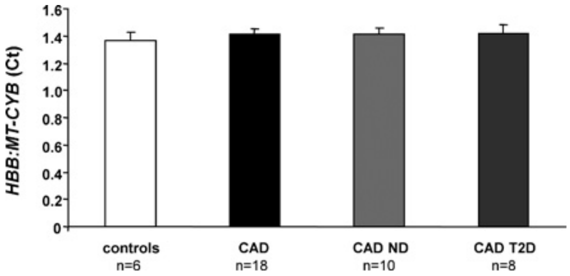

Figure S3 Determination of mitochondrial number in HSVECs by PCR

Total DNA was isolated from HSVECs using the QIAamp® DNA Mini Kit (Qiagen) and 25 ng used for PCR reactions. Using TaqMan® Gene Expression Assays (Applied Biosystems) for single copy nuclear *HBB* and *MT-CYB*, abundance of mitochondrial DNA relative to nuclear DNA was determined by calculating C_t (cycle threshold value) ratios for each patient. No difference in C_t ratio between patient groups was observed, indicating HSVEC mitochondrial numbers do not vary across the groups. CAD ND, CAD patients without T2D; CAD T2D, CAD patients with T2D.

© 2013 The Author(s)
The author(s) has paid for this article to be freely available under the terms of the Creative Commons Attribution Licence (CC-BY) (http://creativecommons.org/licenses/by/3.0/) which permits unrestricted use, distribution and reproduction in any medium, provided the original work is properly cited.

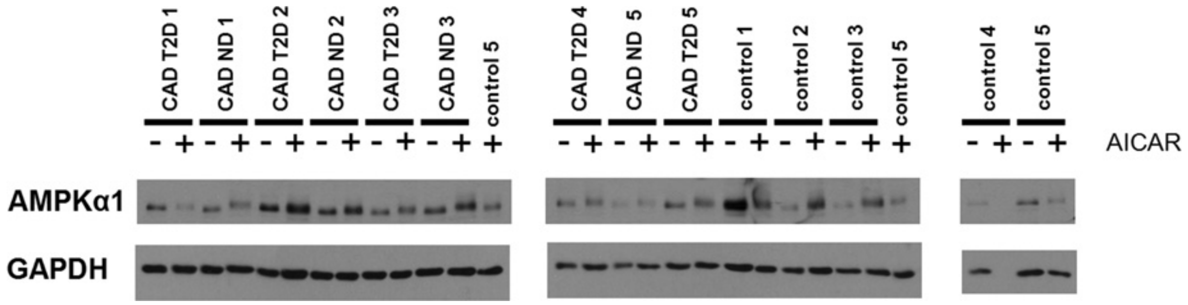

Figure S4 **AMPKα1 expression in HSVECs**
Cells were incubated in the presence (+) or absence (−) of AICAR (2 mmol/l), an artificial ROS-independent activator of AMPK known to stimulate the kinase in intact cells [1]. Lysates were then prepared from HSVECs of control subjects ($n = 5$), and CAD patients without T2D (CAD ND, $n = 5$) and with T2D (CAD T2D, $n = 5$) in the same way as described previously [2]. Lysates were subjected to Western blotting and probed with sheep anti-AMPK-α1 antibody (1:1000 dilution) before incubation with donkey anti-rabbit IgG (1:1000 dilution; GE Healthcare). Resultant immunoblots demonstrated no change in AMPK-α1 expression between patient groups under basal conditions or conditions stimulating AMPK phosphorylation.

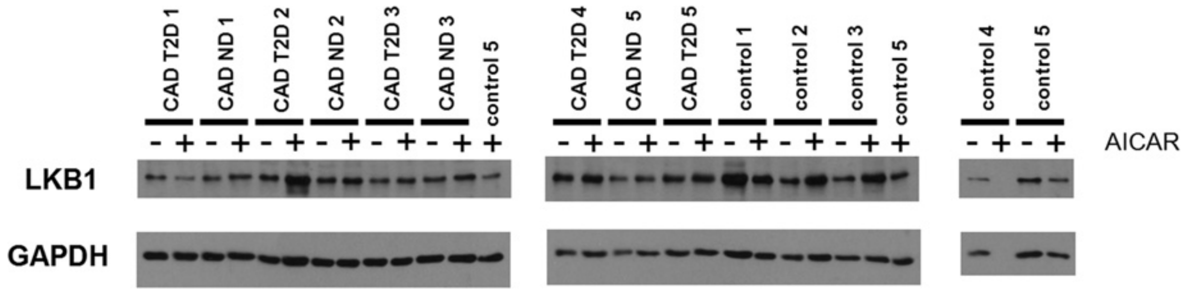

Figure S5 **LKB1 expression in HSVECs**
Cells were incubated in the presence (+) or absence (−) of AICAR (2 mmol/l)) and lysates prepared from HSVECs of control subjects ($n = 5$), and CAD patients without T2D (CAD ND, $n = 5$) and with T2D (CAD T2D, $n = 5$) in the same way as described previously [2]. Lysates were subjected to Western blotting and probed with sheep anti-LKB1 antibody (1:500 dilution) before incubation with protein G-peroxidase from *Streptococcus* sp. (1:1000 dilution; Sigma). Resultant immunoblots demonstrate no change in LKB1 activity between patient groups under basal conditions or conditions stimulating AMPK phosphorylation.

© 2013 The Author(s)
The author(s) has paid for this article to be freely available under the terms of the Creative Commons Attribution Licence (CC-BY) (http://creativecommons.org/licenses/by/3.0/) which permits unrestricted use, distribution and reproduction in any medium, provided the original work is properly cited.

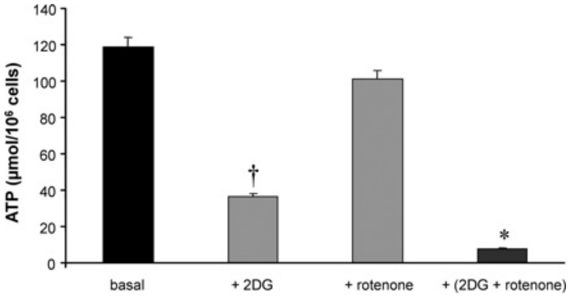

Figure S6 ATP production in HSVECs
Determination of the contribution of oxidative phosphorylation and glycolysis to the generation of ATP in HSVECs was carried out in Professor Moncada's laboratory, using the method described by Quintero and co–workers [3]. HSVECs isolated from CAD patients were grown for 24 h in 96-well plates (PerkinElmer 3603 clear bottom, black walls, seeding density 10000 cells per well) in phenol-red free ECM2 supplemented with 2 % (v/v) FBS. On the day of the experiment, fresh medium was added and cells pretreated with 20 mmol/l 2DG and 0.5 μmol/l rotenone (both Sigma). 2DG is a glycolytic pathway inhibitor and rotenone, an inhibitor of the mitochondrial respiratory chain [3]. ATP was measured by the luciferin/luciferase method with the PerkinElmer ATPlite^{TM} Luminescence Assay System, following the manufacturer's protocol. Chemiluminescence was determined in a TopCount (Packard Biosciences). The addition of 2DG causes a greater decrease in intracellular ATP production than the addition of rotenone and there is an additive effect when inhibitors are used in combination. Results support findings that endothelial cells are highly glycolytic [3]. *and †$P < 0.0001$ relative to basal values for untreated cells.

REFERENCES

1 Corton, J. M., Gillespie, J. G., Hawley, S. A. and Hardie, D. G. (1995) 5-aminoimidazole-4-carboxamide ribonucleoside. A specific method for activating AMP-activated protein kinase in intact cells? Eur. J. Biochem. **229**, 558–565

2 Hardie, D. G., Haystead, T. A. J., Salt, I. P. and Davies, S. P. (1999) Assay and purification of protein: serine/threonine kinases. Protein Phosphorylation: A Practical Approach 2nd edn, (Hardie, D. G., ed.), pp. 201–209, Oxford University Press, Oxford

3 Quintero, M., Colombo, S. L., Godfrey, A. and Moncada, S. (2006) Mitochondria as signaling organelles in the vascular endothelium. Proc. Natl. Acad. Sci. U.S.A. **103**, 5379–5384

Received 9 May 2012/11 October 2012; accepted 11 October 2012
Published as Immediate Publication 11 October 2012, doi: 10.1042/CS20120239

© 2013 The Author(s)
The author(s) has paid for this article to be freely available under the terms of the Creative Commons Attribution Licence (CC-BY) (http://creativecommons.org/licenses/by/3.0/) which permits unrestricted use, distribution and reproduction in any medium, provided the original work is properly cited.
